# Biomedical Membrane of Fish Collagen/Gellan Gum Containing Bone Graft Materials

**DOI:** 10.3390/ma15082954

**Published:** 2022-04-18

**Authors:** Jin Kim, Chang-Moon Lee, Seong-Yong Moon, Young-IL Jeong, Chun Sung Kim, Sook-Young Lee

**Affiliations:** 1 Department of Oral and Maxillofacial Surgery, Chosun University Dental Hospital, Gwangju 61452, Korea; cream4251@hanmail.net (J.K.); msygood@chosun.ac.kr (S.-Y.M.); 2 School of Healthcare and Biomedical Engineering, Chonnam National University, Yeosu 59662, Korea; cmlee@jnu.ac.kr; 3 Department of Biomedical Engineering, Chonnam National University, Yeosu 59662, Korea; 4 Research Center of Healthcare and Biomedical Engineering, Chonnam National University, Yeosu 59662, Korea; 5 The Institute of Dental Science, Chosun University, Gwangju 61452, Korea; yangjuil@outlook.kr; 6 Department of Oral Biochemistry, College of Dentistry, Chosun University, Gwangju 61452, Korea; 7 Marine Bio Research Center, Chosun University, Wando 59146, Korea

**Keywords:** fish collagen, gellan gum, bone graft materials, membrane

## Abstract

The development of a guided bone regeneration (GBR) membrane with non-mammalian fish collagen has the advantage of low risk for transmission of infectious diseases in tissue regeneration. In this work, a fish collagen/gellan gum and bone graft material (FC/GG-BGM) composite GBR membrane were fabricated through solution blending and casting procedures in a vacuum. The membranes were characterized using Fourier transform infrared spectroscopy (FT-IR), X-ray diffraction (XRD), scanning electron microscopy observation (SEM), and atomic force microscope (AFM) analyses. FT-IR results suggested that ionic interactions were formed between FC and GG both in composite powder and membranes. In vivo experiments showed that these FC/GG-BGM composite membranes could generate osteoblast minerals and promote loose bone calcification, thus accelerating bone regeneration. At 2 weeks, the defected site of rats treated with FC/GG-BGM membrane (0.377 ± 0.012 mm^3^) showed higher regeneration than that of rats treated with the bovine collagen membrane (0.290 ± 0.015 mm^3^) and control rats without membrane (0.160 ± 0.008 mm^3^). Compared with bovine collagen membrane, the FC/GG-BGM composite membrane displays better bone regeneration ability. Therefore, FC/GG-BGM composite membrane is suitable as a GBR membrane for bone regeneration.

## 1. Introduction

Bone defects may arise as a consequence of a congenital defect or may be caused by severe trauma, osteomyelitis, tumor growth, or tumor resection [1,2,3,4,5]. Critical-sized bone defects are unable to heal voluntarily. They require reconstruction surgery [3,4,5]. Guided bone regeneration (GBR) is a current treatment option for repairing bone defects [6,7,8,9]. GBR, which is a dental surgical procedure, requires barrier membranes to organize the growth of gingival tissue and bone at the site of bone defects. The function of barrier membranes for GBR is to prevent apical migration of fibroblasts to the site of bone defects and to allow further growth of bone progenitor cells [9,10]. To prepare for successful GBR, it is important to use biocompatible materials and have proper morphological architecture for barrier membranes. Investigating bone substitutes remains an ongoing frontier field [6,7,8,9,10,11,12]. Dimitriou et al. argued that ideal barrier membranes and bone grafting materials should be established to mimic the normal process of bone formation [9]. From these points of view, various kinds of materials including non-degradable and degradable polymers have been developed for successful GBR in the last few decades [9,10,11,12,13,14]. Among them, biodegradable polymers can avoid secondary surgical treatment after bone regeneration [6,13,14,15]. In recent, Tunthasen et al. have reported that a semi-rigid shell barrier system made of polycaprolactone (PCL) and biphasic calcium phosphate can serve as a reasonable guide membrane having good physicomechanical properties without showing toxicity to osteoblast or fibroblast cells [13]. However, despite studies using many biodegradable polymers, some drawbacks to the development of the polymer-based GBR membranes remain to be solved. For example, the fast degradation rate, biological response in the microenvironment, and poor mechanical strength of the biodegradable polymers are still limiting their applications in GBR [6].

Among various biomaterials, collagen as a connective tissue component has been spotlighted for application in bioresorbable membranes due to its excellent biocompatibility, resorbability, cell-friendly properties, ability to provide structural support, and low immunogenicity [10,11,16,17]. From these viewpoints, many scientists have investigated collagen as a biomaterial for successful GBR [10,11,16,17,18]. For example, Liang et al. have reported that 4-vinylbenzyl chloride-functionalized photoactive atelocollagen shows superior proteolytic stability in vitro and tissue formation in vivo to commercial dental membranes [10]. In the medical field, bovine collagen as a scaffold for tissue engineering has been widely used. However, because of the risk of transmission of bovine spongiform encephalopathy to human beings, the use of bovine collagen has been reconsidered [19]. Recently, fish collagen from shark and salmon skin is receiving a lot of attention because of the low risk for transmission of infectious diseases [20]. Despite these advantages of fish collagen, this material still requires modification to achieve effective outcomes in the clinic, provide appropriate physicomechanical support for bone regeneration, and show osteoinductive activity in bone defects [16]. For these purposes, collagen is frequently blended with other biomaterials, such as chitosan, pectin, and hyaluronic acid (HA) to improve its physical/biological performance [16,17,18]. For example, an HA-collagen scaffold can lead to more new bone formation in critical size defects than a collagen scaffold [18].

Recent studies have paid significant attention to the development of GBR membranes loaded with bone graft materials (BGM) to be utilized in defect areas for promoting bone regeneration [21,22,23,24,25]. Granules of BGM are mixed with autologous blood to form a paste, which can be contoured as needed and then loaded with GBR membranes to regenerate bone efficiently [25,26,27]. Bone-based synthetic graft biomaterials for tissue engineering treatments are being explored for better treatment of bone defects and bone-related diseases [23,24,25,26,27]. For these purposes, numerous techniques and biomaterials have been developed for orthopedic treatment over the past several decades [23,24,25,26]. Fushimi et al. have reported that hydrogel-based graft materials composed of recombinant polypeptide based on the human type I collagen alpha 1 chain (RCPhC1) can be fabricated through scalable streamlined production procedure, resulting in robust regeneration of mature bone tissues in pre-clinical animal models [27].

The aim of this study was to fabricate a BGM-loaded GBR membrane using fish skin-derived collagen (FC) and gellan gum (GG) for application in guided bone and bone regenerative therapy. GG used for fabricating GBR membranes has non-toxicity, biocompatibility, biodegradability, and transparency of its hydrogel [28]. Furthermore, GG has excellent potency as a scaffold material for cartilage tissue application [28,29]. A BGM-loaded FC/GG membrane (FC/GG-BGM membrane) was prepared and characterized both in vitro and in vivo for bone regeneration. In particular, in order to compare the bone regeneration ability of the FC-GG-BGM membrane, it was evaluated by comparing the regeneration with commercial bovine collagen. The morphology, structure, cytocompatibility, and bone regeneration ability of the BGM-loaded GBR membrane were then investigated.

## 2. Materials and Methods

### 2.1. Materials

Fish collagen (FC, Mw 5000 Da) was purchased from AFC-HD AMS Life science Co., Ltd. (Shizouka, Japan). Gellan gum (GG, Gelzan^CM^ (Sigma-Aldrich Co., St. Louis, MO, USA), Mw 1000 kDa), D-PBS (calcium chloride, magnesium chloride, pH 7.4), ascorbic acid, naphthol AS-BI phosphate, β-glycerolphosphate, dexamethasone (DEX), ethylenediaminetetraacetic acid (EDTA), citrate-buffered acetone, alkaline-dye mixture, Mayer’s hematoxylin solution, and 3-(4,5-Dimethylthiazol-2-yl)-2,5-Diphenyltetrazolium Bromide (MTT) were obtained from Sigma-Aldrich Co. (St. Louis, MO, USA) and Invitrogen Co. (Waltham, MA, USA), respectively. Bone graft materials (BGM) was purchased from Geistlich Pharma AG (Wolhusen, Switzerland). Fetal bovine collagen membrane (Lyostypt^®^) was purchased from B. Braun (Melsungen, Germany). All other reagents were purchased from Sigma-Aldrich Co. (St. Louis, MO, USA) unless otherwise specified.

### 2.2. Preparation of FC/GG and FC/GG-BGM Membranes

**Figure 1:**
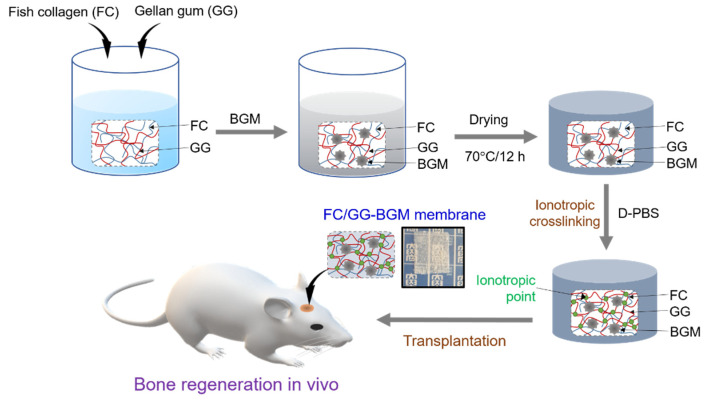
Schematic illustration for bone regeneration in vivo using a biomedical membrane made of fish collagen (FC)/gellan gum (GG) containing bone graft materials (BGM).

FC and GG were dissolved in 100 mL of deionized water (DW) with concentrations from 2% (*w/v*) at mass ratios of 1:1 (*w/w*) and heated at 85–90 °C until they became transparent solutions. FC/GG membranes containing BGM (100 mg) were prepared as follows. BGM was uniformly dispersed in FC/GG solution at 50 °C. These solutions were poured into Petri dishes (90 mm × 15 mm) and evaporated at 70 °C for 12 h to dry membranes. FC/GG and FC/GG-BGM membranes were then cross-linked by immersing them in D-PBS for 24 h at room temperature. These cross-linked membranes were then washed with DW three times to remove residual D-PBS and then dried at room temperature. After crosslinking, membranes were referred to as FC/GG and FC/GG-BGM, respectively (Figure 1).

### 2.3. Characterization of the FC/GG-BGM Membrane

Surfaces and cross-sectional microstructures of BGM, FC/GG, and FC/GG-BGM membranes were examined with a scanning electron microscope (SEM, S-4700, HITACHI, Tokyo, Japan). Before SEM observation, all dried samples (5 × 5 mm) were sputter-coated with gold. SEM imaging was carried out in the low-vacuum mode at an accelerating voltage of 15 kV. Thicknesses of FC/GG and FC/GG-BGM membranes were measured using a digital micrometer (MDC-25 SB, Mitutoyo Co., Tokyo, Japan). Chemical groups in FC/GG and BGM were determined via Fourier-transform infrared spectroscopy (FT-IR). Infrared spectra were obtained using an AIM-9000 model (Shimadzu Co., Ltd., Kyoto, Japan) to confirm the preparation of the FC/GG-BGM membrane. X-ray diffraction patterns of BGM, FC/GG membrane, and FC/GG-BGM membrane were recorded using an X-ray diffractometer (XRD, Rigaku D/Max Ultima III, Tokyo, Japan) operating at 40 kV with 40 mA at a scan rate of 2°/min. The Cu Kα X-ray was filtered with nickel. Atomic force microscopy (AFM) was performed with an atomic force microscope (Park XE-15, Park Systems Corp., Suwon, South Korea) to determine the surface roughness, morphology, and dispersion of BGM.

### 2.4. Measurement of Mechanical Properties

FC/GG and FC/GG-BGM membranes were cut into 3 × 1 cm^2^ pieces. To determine the mechanical properties of these FC/GG-BGM membranes, mechanical parameters were recorded automatically using a universal test machine (UTM, TO-102, Testone Co., Siheung, South Korea) at a crosshead speed of 5 mm/min.

### 2.5. Alkaline Phosphatase (ALP) Activity

To analyze MC3T3-E1 cell differentiation on FC/GG membranes and FC/GG-BGM membranes, activities of ALP and calcification were examined after a 14 days incubation in the aforementioned culture medium supplemented with 50 μg/mL ascorbic acid, 10 mM β-glycerolphosphate, and 10^−8^ M dexamethasone. The ALP activity was visualized using naphthol AS-BI phosphate with fast red violet and sodium citrate as couplers according to the manufacturer’s protocol. In brief, cultured cells grown on FC/GG membranes and FC/GG-BGM membranes were fixed by immersion in citrate-buffered acetone for 30 s, rinsed with deionized water, immersed in an alkaline-dye mixture at room temperature for 30 min, rinsed with DW, counterstained with Mayer’s hematoxylin solution. They were then investigated microscopically and photographed.

### 2.6. In Vitro Cytotoxicity Assay

The cytotoxicities of membranes were assayed in vitro using an MTT kit. L292 cells (a mouse fibroblast cell line) were cultured in Dulbecco’s modified Eagle’s medium supplemented with 10% fetal bovine serum and 1% antibiotics in 5% CO_2_ at 37 °C. These cells were placed in a 96-well plate at a density of 1 × 10^4^ cells/well. The medium was replaced with fresh medium containing various concentrations of the membrane extraction solution, which was prepared in accordance with the guidance of ISO 10993. Samples were immersed in PBS (0.1 M, pH 7.4) at 37 °C for 24 h, after which the cell culture was mixed with an MTT assay reagent for 4 h and then read on a multi-well microplate reader (BioTek Instruments Inc., Winooski, VT, USA) at 570 nm.

### 2.7. Experimental Animal Model and Surgical Procedure

To investigate bone regeneration behavior of FC/GG-BMG membranes, a skull defect model of Sprague-Dawley (SD) rats (Female, 250 g, age 8 weeks, Orient, Suwon, Korea) was used. All procedures involving animals were approved by the Institutional Animal Care Committee of the Research Institute of Medical Science at Chosun National University (No: CIACUC2019-S0019). Animals were anesthetized by intraperitoneal injection of a mixture of alfaxalone and rompun (1:1) (0.1 mL/100 g). Following the injection, the skull was shaved and surfaces surrounding the skull were exposed via a full-thickness incision followed by an injection of 2% lidocaine with 1:100,000 epinephrine the parietal scalp of each rat. A man-made light defect was generated with an 8 mm dental round burr. All scalp defect sites were filled with BGM and then covered with the membranes of bovine collagen membrane, FC/GG membrane, or FC/GG-BGM membrane, respectively. The experimental animal model was divided as follows. There were four groups, including a negative control group (A group inserted with only BGM without membrane), a positive control group (B group covered with the bovine collagen membrane), and two experimental groups (C group covered with FC/GG membrane; and D group covered with FC/GG-BGM). Prior to implantation at the defect site, the membrane was hydrated in physiologic saline to restore its elasticity. Animal experiments for bone regeneration were conducted for 2 weeks after the membrane was covered in the scalp defect site (*n* = 3/group). Experimental animals were euthanized by CO_2_ at 2 weeks post-surgery to acquire cranial specimens.

### 2.8. Micro-Computed Tomography (Micro-Ct) Analysis

In each group, four animals were imaged following anesthesia induction and maintenance with 2% isoflurane on a quantum GX μCT imaging system (PerkinElmer, Hopkinton, MA, USA) located in the Korea Basic Science Institute. The exposure parameters were set to levels of 90 kV and 88 μA with a field of view of 45 mm (voxel size: 90 μm, total scanning time: 20 min). Micro-CT images were visualized via a 3D-Viewer (PerkinElmer, Hopkinton, MA, USA) using software accompanying the Quantum GX microsystem. The following procedure was performed for quantitative analysis of a new bone. A 5 × 5 × 5 median filter and a spatial filter module were used to reduce image shaking and to improve the sharpness of the three-dimensional image using an Analyze software 12.0 (Analyze Direct, Overland Park, KS, USA). The region of interest module was used to calculate the volume of the new bone around each bone defect. Finally, a 3D rendering of heterogeneous bone growth was generated. Data of bone volumes were expressed as mean ± standard deviations (S.D.). In each group, four animals were euthanized by CO_2_ after surgery

### 2.9. Histological Preparation and Evaluation

Tissue specimens were decalcified in 17% EDTA solution dehydrated in an ascending graded series of alcohol and embedded in paraffin. A series of 5 μm transverse sections in the center of bone defects or the membrane with soft tissue were prepared and stained with hematoxylin and eosin (H&E) for observation by light microscopy (BX51, Olympus, Tokyo, Japan).

### 2.10. Statistical Analysis

All quantitative data were collected and represented as average ± standard deviation (SD) from at least three measurements for each condition. Differences between groups were analyzed using Student *t*-tests and one-way ANOVA analysis followed by Tukey tests. The differences were considered to be statistically significant at * *p* < 0.05.

## 3. Results

### 3.1. Morphology and Mechanical Properties of FC/GG-BGM Membrane

**Figure 2:**
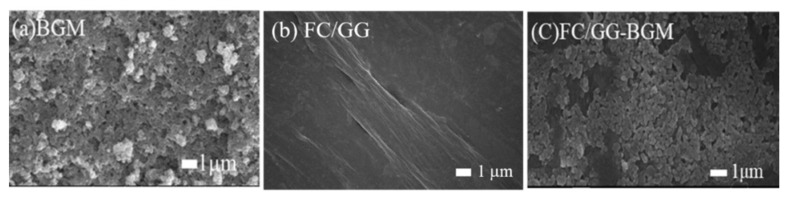
Scanning electron microscopic images for surface morphological observation of (**a**) pure BGM particles, (**b**) FC/GG membrane, and (**c**) FC/GG-BGM membrane.

We prepared FC/GG membranes and FC/GG-BGM membranes. The thickness of all FC/GG membranes measured with a caliper ranged from 10 μm to 30 μm. Surface morphologies of BGM and membranes were observed by SEM. Morphological attributes of BGM particles showed nearly spherical shapes with sizes ranging from approximately 0.5 μm to 1 μm (Figure 2a). Surface morphologies of FC/GG membranes and FC/GG-BGM membranes are shown in Figure 2b and c. BGM particles were observed on the surface of the FC/GG-BGM membrane as expected (Figure 2c). The tensile strengths of the bovine collagen membrane, FC/GG membrane, and FC/GG-BGM membrane were 4.03 ± 1.24, 7.02 ± 0.7, and 5.26 ± 0.88 MPa, respectively. The tensile strength of the FC/GG membrane was higher than those of the other membranes.

### 3.2. FTIR Analysis of the FC/GG-BGM Membrane

**Figure 3:**
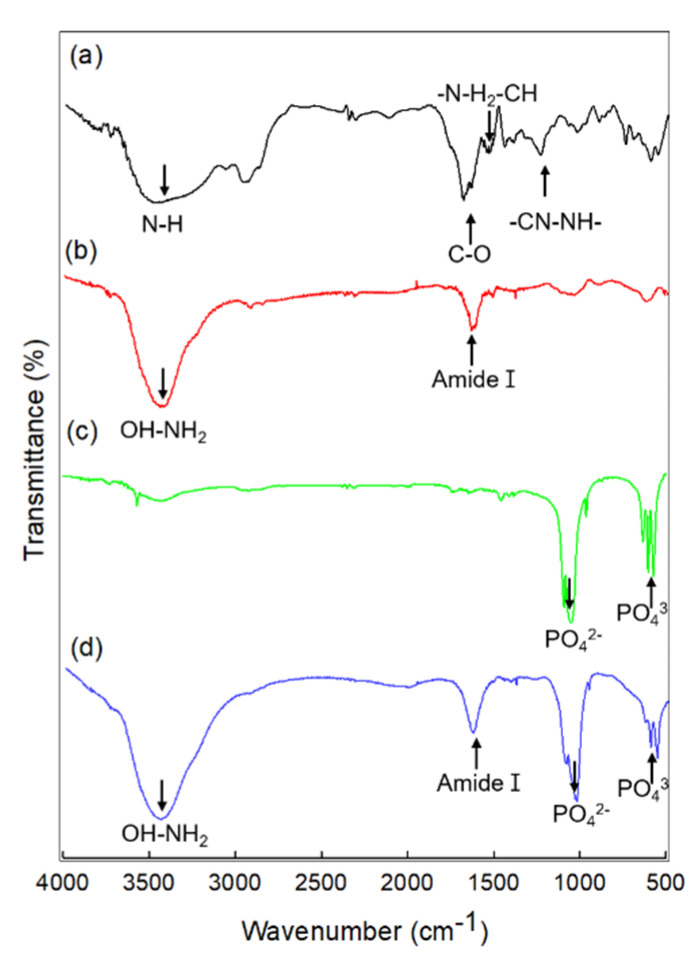
FTIR spectra of (**a**) FC, (**b**) pure BGM, (**c**) FC/GG membrane, and (**d**) FC/GG−BGM membrane.

FTIR spectra for the prepared membranes are shown in Figure 3. The FC spectrum revealed absorption bands at 3480 cm^−1^ (NH stretching), 1630 cm^−1^ (amide I, CO stretching), 1540 cm^−1^ (amide II, NH bending and CH stretching), and 1230 cm^−1^ (amide III, CN stretching and NH bending). The blended FC/GG spectrum showed absorption bands at 1600 cm^−1^ (asymmetric COO^−^ stretching), 1410 cm^−1^ (symmetric COO^−^ stretching), and 1031 cm^−1^ (polysaccharide ring). The peak of absorption bands at around 3400 cm^−1^ (contributions of OH and NH_2_ stretching) also appeared. The peak shifted to lower wavenumbers due to the dehydration of polymer chains. Results suggested a noncovalent interaction between FC and GG. In the BGM spectra, phosphate bands were evident between 900 and 1200 cm^−1^ (PO_4_^3−^ mode at 962 cm^−1^ and PO_4_^3−^ mode at 1026 cm^−1^). These results demonstrated the presence of interactions among all components of the blend. In particular, the shift of FC/GG confirmed the presence of interactions among C=O of the protein chain and calcium ions of BGM. The shift of the band due to COO^−^ of GG demonstrated the presence of ionic interactions between FC and polysaccharides.

### 3.3. XRD of the FC/GG-BGM Membrane

**Figure 4:**
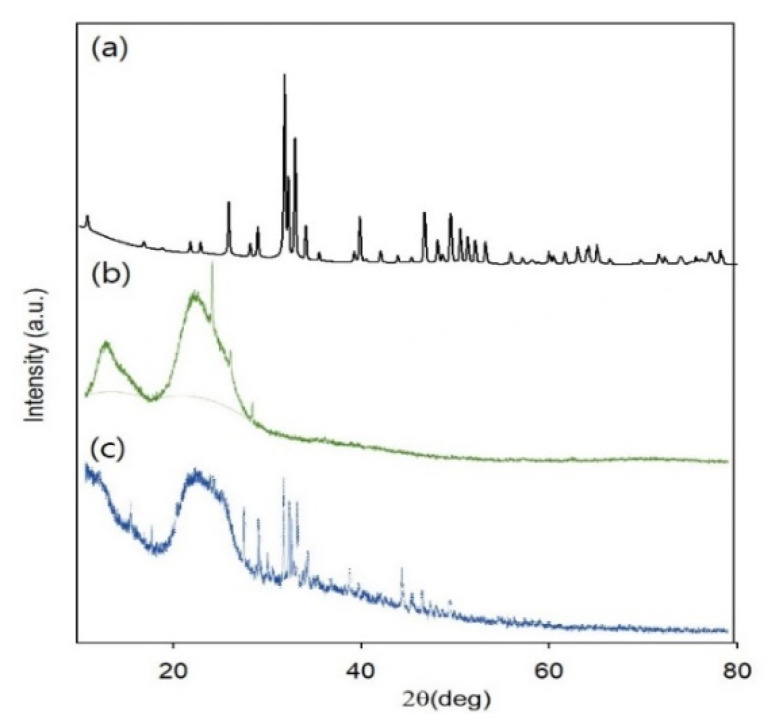
XRD patterns of (a) pure BGM, (b) FC/GG membrane, and (**c**) FC/GG-BGM membrane.

Figure 4 shows X-ray diffraction patterns of pure BGM, FC/GG membrane, and FC/GG-BGM membrane. FC/GG membrane showed a large amorphous hump at 2θ of 15 to 30 [11]. In X-ray diffraction patterns of FC/GG-BGM membrane, peaks were subdued as compared to much highly crystalline BGM peaks. The weak crystalline nature of the FC/GG-BGM membrane as compared to BMG particles was due to the presence of a predominantly amorphous FC/GG phase in the membrane as shown in Figure 4.

### 3.4. AFM and Mechanical Analysis of the FC/GG-BGM Membrane

**Figure 5:**
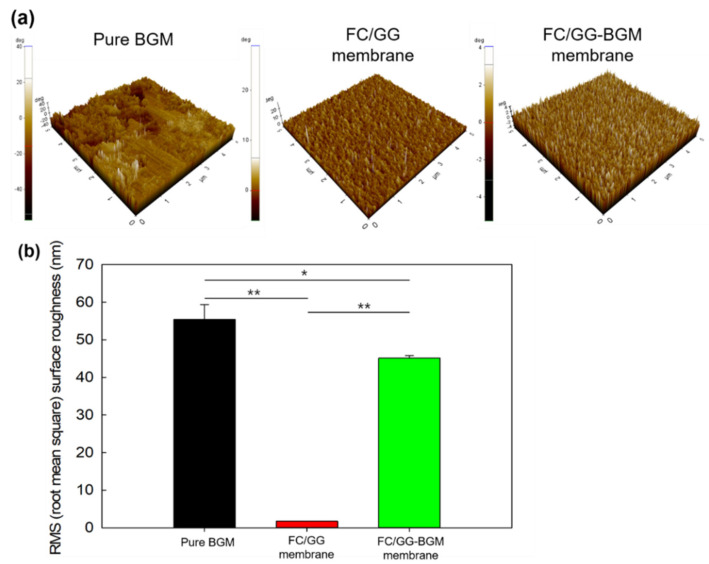
(**a**) 3D surface images of pure BGM, FC/GG membrane, and FC/GG-BGM membrane. Surface morphology and profile observed at three points (5 μm × 5 μm area per point) of each sample using AFM. (**b**) Surface roughness (below graph) of pure BGM, FC/GG membrane, and FC/GG-BGM membrane calculated from the value of the surface profile using the root mean square formulation. Data are reported as the mean ± SD of three replicates. (* *p* < 0.05, ** *p* < 0.01).

The surface roughness and topographic analyses of pure BGM, FC/GG membrane, and FC/GG-BGM membrane were evaluated by AFM. The AFM images and surface roughness values of pure BGM, FC/GG membrane, and FC/GG-BGM membrane are shown in Figure 5. It was found that the FC/GG membrane was almost smooth (Figure 5b). The root mean square (RMS) value of the FC/GG membrane was 1.07 ± 0.02 µm. On the other hand, the surface of the FC/GG-BGM membrane became visibly rough (Figure 5a). When BGM crystals were added to the membrane, the RMS value increased to 45.83 µm. These results indicate that the addition of BGM into the FC/GG membrane resulted in an enhancement of surface roughness (Figure 5b).

### 3.5. Cytotoxicity of the FC/GG-BGM Membrane

**Figure 6:**
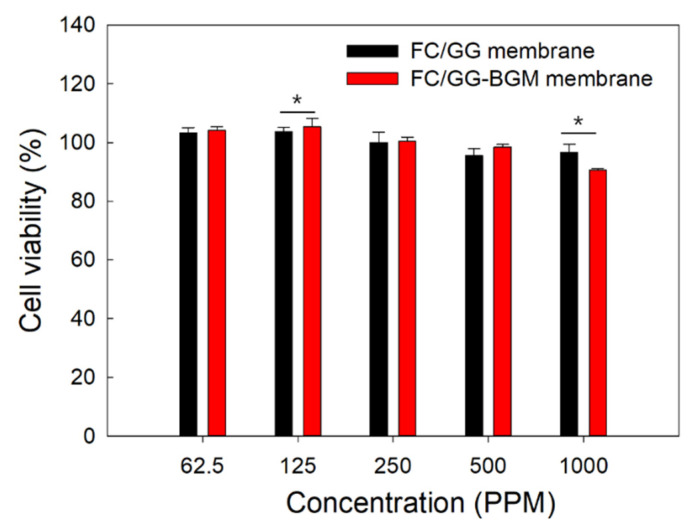
Cell viabilities of L929 cells treated with the extracted solution from the FC/GG membrane or the FC/GG-BGM membrane at various concentrations. (* *p* < 0.05).

Cell viability was determined using an MTT assay. L929 cells were treated with the extracts of FC/GG membrane or FC/GG-BGM membrane at different concentrations (62.5 ppm, 125 ppm, 250 ppm, 500 ppm, and 1000 ppm) (Figure 6). As expected, the extracts of the FC/GG membrane and FC/GG-BGM membrane did not affect the growth of L929 cells despite the high concentration.

### 3.6. ALP Activity of the FC/GG-BGM Membrane

**Figure 7:**
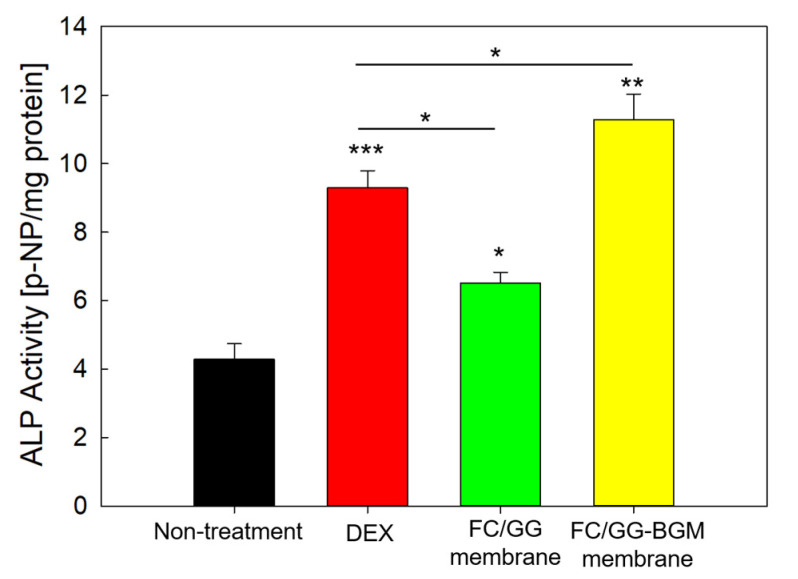
ALP activity of MC3T3-E1 cells cultured on FC/GG membranes and FC/GG-BGM membranes for 14 days (* *p* < 0.05, ** *p* < 0.01, *** *p* < 0.001). DEX means dexamethasone and was used as a positive control.

ALP is a hydrolase enzyme that is elevated during osteoblast differentiation. Thus, we measured ALP activity after culturing MC3T3-E1 cells on FC/GG membranes and FC/GG-BGM membranes for 14 days. As shown in Figure 7, MC3T3-E1 cells cultured on FC/GG-BGM membranes displayed significantly higher ALP activity than the FC/GG membranes (* *p* < 0.01). This observation indicates that FC/GG-BGM membranes have a greater osteogenic effect on MC3T3-E1 cells than that FC/GG membranes in vivo. Surface interactions between FC/GG-BGM membrane and cells play important roles in the formation of surface complexes on bone mineral, their biorecognition, osteoinductance, and osteoconductance.

### 3.7. Micro-CT Imaging

**Figure 8:**
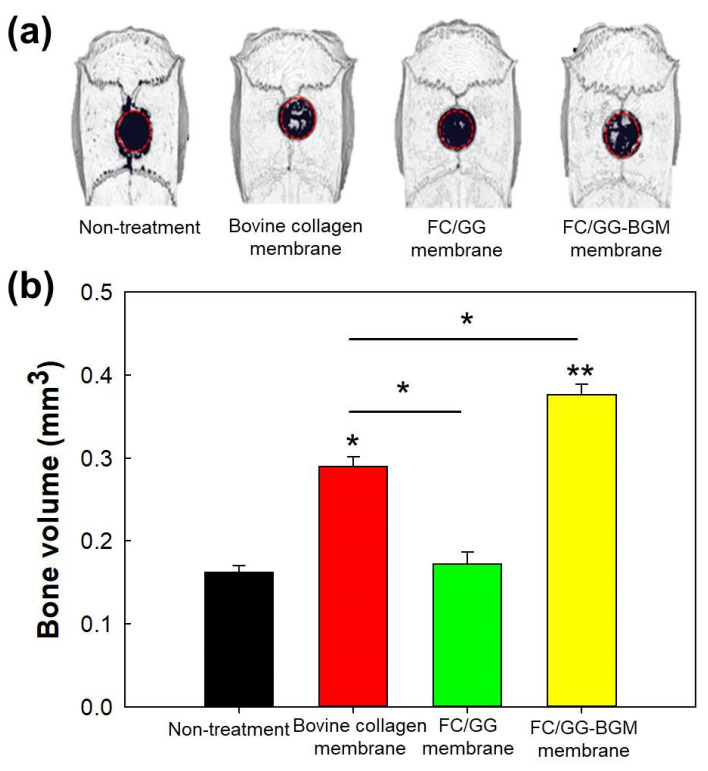
(**a**) Representative micro-CT image and (**b**) evaluation of the bone volume (below graph) of defects treated with bone grafts materials and membranes (non-treatment, bovine collagen membrane, FC/GG, FC/GG-BGM membrane) after 2 weeks. (* *p* < 0.05, ** *p* < 0.01).

Reconstructed three-dimensional images of the rat’s skull obtained from micro-CT showed a result similar to that from ALP activity analysis (Figure 8). A three-dimensional image was obtained with an automatically set-up threshold level. The bone regeneration of the defect site of the rat covered with FC/GG-BGM membrane at 2 weeks was greater than that in other groups.

### 3.8. Histological Examination

**Figure 9:**
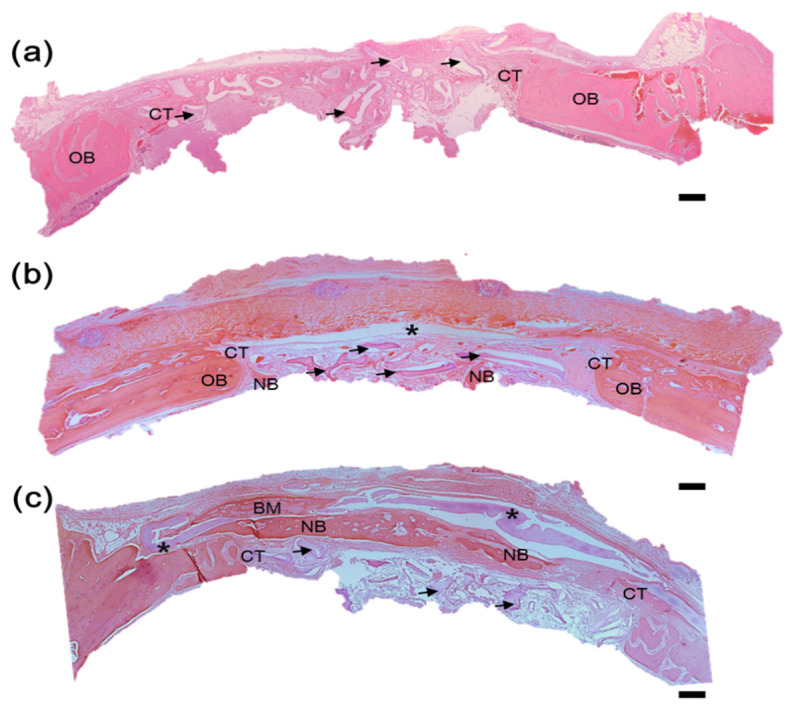
Histological sections stained with H&E show the cranial defect and surrounding cranial tissue, (**a**) covered without membrane, (**b**) covered with collagen membrane (asterisk), and (**c**) covered with FC/GC-BGM membrane (asterisk) after 2weeks (OB: original bone, CT: connective tissue, NB: newly formed bone, BM: bone marrow, BGM: black arrow). Scale bar indicates 100 μm.

All animals were clinically healthy, presenting only slight edema in the operating region, which was expected after the surgical procedure was performed. Neither group presented any signs of necrosis, suppuration, or infection. Two weeks after surgery, it was observed that FC/GG-BGM was well combined with the defect tissue (Figure 9c). For the commercial bovine collagen membrane, H&E staining showed no presence of inflammatory cells in the membrane (Figure 9b). No signs of postoperative infection or exposure to the membrane were observed at two weeks postoperatively. New bone formation was integrated with old bone areas close to the defect edge in most samples. The FC/GG-BGM membrane group did not exhibit fibrous tissue invasion under the membrane. Newborn results indicated that integration in the FC/GG-BGM group occurred at two weeks (Figure 9c). In the positive control group, the bone had mostly degenerated at two weeks. Membranes in the FC/GG-BGM group were mostly preserved with their morphologies retaining their original form (Figure 9c). The FC/GG-BGM group also showed the formation of blood vessels and new bone upon histological evaluation.

## 4. Discussion

Barrier membranes should fulfill the specific properties demanded by GBR applications, i.e., membranes for GBR require appropriate physicomechanical properties, low immunogenicity, biocompatibility with surrounding tissues or cells, and cell-embedding properties, and ease of usability in the clinics [17,18]. Because collagen fulfills most of these requirements, it has been widely investigated as a barrier membrane for the fabrication of membranes and for successful GBR [10,11,12,18,19,20,26,27]. Collagen is known to promote hemostasis by stimulating platelet attachment and fibrin linkage increase [18,30,31]. These properties of collagen provide an appropriate surface for osteoblast attachment, thus supporting osteogenesis [31]. Furthermore, these superiorities enable collagen to apply as a scaffold for neovascularization and chemoattractant for fibroblasts [18,30,31].

Due to these reasons, collagen sourced from bovine and porcine sources has been extensively utilized in biomedical applications. However, it has the potential risks of disease transmission to humans [19]. On the other hand, collagen extracted from fish skin is believed to be a safer and cheaper candidate for use in humans. Thus, it has been spotlighted in the bioindustry [32,33,34]. Sugiura et al. [34] have reported that collagen from tilapia shows similar physicomechanical properties and bioresorption properties to porcine collagen. For these reasons, FC has been investigated as a scaffold or carrier for tissue engineering [32,33,34]. However, collagen itself is still needed to be modified with other functional materials to improve its physicomechanical potential for application in GBR. Thus, we fabricated a BGM-loaded membrane using FC and GG for this study.

GG is composed of repeating carbohydrates including two D-glucose, one L-rhamnose, and one D-glucuronic acid. It is an anionic linear microbial exopolysaccharide (EPS). GG is considered a suitable candidate for cartilage tissue scaffold because it is known to possess outstanding properties as a biomaterial, such as non-toxicity, biocompatibility/biodegradability, and transparency of its hydrogel [35]. Our group has previously prepared scaffolds composed of croaker swim bladder-derived collagen and GG for GBR membrane with the ability to augment bone regeneration [36]. In this study, FC/GG-BGM membranes showed appropriate biocompatibility and bone formation ability, i.e., cell viability was not significantly changed between FC/GG membranes and FC/GG-BGM membranes as shown in Figure 6. Furthermore, ALP activity of FC/GG-BGM membranes was significantly increased as shown in Figure 7, indicating that FC/GG-BGM membranes had superior potential in osteoblast differentiation. Micro-CT results also indicated the higher potency of FC/GG-BGM membranes in the degree of defect fill as shown in Figure 8 and Figure 9. As a geometrical configuration, the surface properties of FC/GG-BGM membranes might influence bone ingrowth and osteogenesis in connection with the activity of bone morphogenetic proteins adsorbed at the interfaces [37,38]. Furthermore, the FC/GG-BGM membrane maintained its intact shape for 14 days at the skull defect site while the commercial natural collagen membrane was mostly degraded (data not shown). These results might be due to the slow degradation rate of the FC/GG-BGM membrane. In addition to the research results presented in this study, it is necessary to further study the degradation pattern of FC/GG-BGM membrane and bone regeneration after 4 weeks in vivo. Although the practical use of FC/GG-BGM membranes should be evaluated for application in clinics, these results suggest that FC/GG-BGM membranes have excellent potency as novel biocompatible/bioactive barrier membranes for GBR in clinical applications.

## 5. Conclusions

FC/GG-BGM membrane was fabricated through solution blending and casting procedures in a vacuum. In vivo experiments show that FC/GG-BGM membrane had the potential to improve osteoblast mineralization and promote loosening of bone calcification, thus accelerating bone regeneration. Compared with the bovine membrane and FC/GG membrane, FC/GG-BGM membranes displayed higher bone regeneration ability. Thus, they could be considered suitable candidates for GBR membranes. Our results suggest that FC/GG-BGM composite membranes are promising candidates for successful GBR. In future research, we plan to confirm the effective bone regeneration of FC/GG-BGM membrane by applying it to various indications.

## Data Availability

The data presented in this study are available in this article.
